# Supplementary material for: On-Treatment Decrease of Serum Interleukin-6 as a Predictor of Clinical Response to Biologic Therapy in Patients with Inflammatory Bowel Diseases
Source: J Clin Med. 2020 Mar 15;9(3):800. doi: 10.3390/jcm9030800 (PMC7141255; doi:10.3390/jcm9030800)
Supplement: Supplementary file 1 [file jcm-09-00800-s001.pdf]

Journal of
Clinical Medicine

Supplementary Materials

Table S1. Comparison of the baseline values of zonulin, soluble CD163 (sCD163), and cytokines between patients with CD and those with UC.

	CD (n = 72)	UC (n = 29)	p-Value
Zonulin (ng/mL), median (95% CI)	45.3 (43.1–48.3)	46.0 (41.7–50.1)	0.559
sCD163 (ng/mL), median (95% CI)	547 (488–627)	547 (472–714)	0.549
IL-6 (pg/mL), median (95% CI)	1.62 (0.90–3.68)	4.56 (2.39–7.16)	0.104
IL-8 (pg/mL), median (95% CI)	5.79 (5.15–9.18)	6.19 (4.39–8.20)	0.548
IL-10 (pg/mL), median (95% CI)	0.01 (0.01–1.61)	0.01 (0.01–1.43)	0.881
IL-33 (pg/mL), median (95% CI)	4.64 (0.01–20.09)	0.01 (0.01–3.04)	0.209
TNFα (pg/mL), median (95% CI)	11.27 (8.32–14.71)	8.77 (6.47–14.21)	0.549

Abbreviations: Crohn's disease (CD), ulcerative colitis (UC), interleukin (IL), interferon (IFN), tumor necrosis factor-alpha (TNFα), confidence interval (CI).

Table S2. Comparison of zonulin, sCD163, and cytokine values measured at baseline between patients with IBD responders and non-responders to treatment.

	Responders	Non-Responders	p-Value
Zonulin (ng/mL), median (95% CI)	44.2 (41.8–48.0)	48.6 (43.3–53.4)	0.563
sCD163 (ng/mL), median (95% CI)	503 (450–560)	552 (465–652)	0.314
IL-6 (pg/mL), median (95% CI)	1.44 (0.90–2.45)	0.90 (0.54–2.81)	0.647
IL-8 (pg/mL), median (95% CI)	6.62 (5.79–10.56)	8.15 (5.09–11.57)	0.976
IL-10 (pg/mL), median (95% CI)	3.56 (0.01–6.05)	1.61 (0.01–3.96)	0.698
IL-33 (pg/mL), median (95% CI)	8.76 (0.07–49.86)	16.87 (0.01–57.87)	0.665
TNFα (pg/mL), median (95% CI)	13.79 (8.75–16.82)	15.60 (7.86–19.97)	0.796

Abbreviations: interleukin (IL), tumor necrosis factor-alpha (TNFα), confidence interval (CI).
